# The Positional-Specificity Effect Reveals a Passive-Trace Contribution to Visual Short-Term Memory

**DOI:** 10.1371/journal.pone.0083483

**Published:** 2013-12-26

**Authors:** Bradley R. Postle, Edward Awh, John T. Serences, David W. Sutterer, Mark D’Esposito

**Affiliations:** 1 Departments of Psychology and Psychiatry, University of Wisconsin-Madison, Madison, Wisconsin, United States of America; 2 Department of Psychology, University of Oregon, Eugene, Oregon, United States of America; 3 Department of Psychology, University of California San Diego, San Diego, California, United States of America; 4 Wills Neuroscience Institute and Department of Psychology, University of California, Berkeley, California, United States of America; University College London, United Kingdom

## Abstract

The positional-specificity effect refers to enhanced performance in visual short-term memory (VSTM) when the recognition probe is presented at the same location as had been the sample, even though location is irrelevant to the match/nonmatch decision. We investigated the mechanisms underlying this effect with behavioral and fMRI studies of object change-detection performance. To test whether the positional-specificity effect is a direct consequence of active storage in VSTM, we varied memory load, reasoning that it should be observed for all objects presented in a sub-span array of items. The results, however, indicated that although robust with a memory load of 1, the positional-specificity effect was restricted to the second of two sequentially presented sample stimuli in a load-of-2 experiment. An additional behavioral experiment showed that this disruption wasn’t due to the increased load per se, because actively processing a second object – in the absence of a storage requirement – also eliminated the effect. These behavioral findings suggest that, during tests of object memory, position-related information is not actively stored in VSTM, but may be retained in a passive tag that marks the most recent site of selection. The fMRI data were consistent with this interpretation, failing to find location-specific bias in sustained delay-period activity, but revealing an enhanced response to recognition probes that matched the location of that trial’s sample stimulus.

## Introduction

Although the visual recognition of objects is remarkably robust to differences in the precise patterns of retinal input produced by an object during its initial vs. subsequent presentations, there are nonetheless conditions under which object recognition performance can benefit when the matching recognition probe is presented in the same retinotopic location as had been the sample item [1]–[3]. Such a positional-specificity effect can be seen when stimuli are novel and nonrepresentational, and when probes are not transformed in any way (e.g., size, rotation, translation) from their associated samples. Dill and Fahle [1] concluded from their studies of this effect that object recognition results from the contributions of feature detectors at multiple levels of the visual system, beginning perhaps in V1 and culminating in inferotemporal (IT) cortex. The positional-specificity effect would presumably arise from contributions by neurons at relatively low levels of the visual system, whose small receptive fields would be sensitive to the precise overlap of repeated presentations of a stimulus. The studies presented in this report use this effect and its theoretical explanation as a tool to address questions of mechanisms underlying short-term and working memory for visual patterns.

Visual short-term memory (VSTM) supports the ability to use recently presented information to guide behavior, even through this information is no longer present in the environment. Many physiological (e.g., [4]–[7]) and computational (e.g., [8]) accounts of VSTM explicitly emphasize a critical role for the active representation of information during the delay period that separates the sample from the stimulus that prompts the behavioral response. We will refer to these as *sustained-activity* models. It is also theoretically plausible, however, that VSTM might also be supported, at least in part, by the creation at encoding of a *passive trace* (e.g., a pattern of synaptic weights) which could then be retrieved at the end of the delay period (e.g., [9], [10]). At least three hypothetical accounts of the positional-specificity effect fall into these two categories. A *sustained-activity* account holds that some of the units in the ventral pathway that are activated during the perception of the sample stimulus maintain an elevated level of activity across the delay period. In this scenario, recognition of a matching recognition probe would be facilitated because its perception, at every station of visual analysis, would engage these same units. Two alternatives appeal to a *passive-trace* mechanism. By a *repetition suppression* account, the perception of an identical item in the same retinal location recruits the same networks that had been engaged in its initial perception, and the reactivation of these networks is facilitated relative to the initial presentation. By a *repetition enhancement* account, the reactivation of these networks prompts an attention-based enhancement of the neural representation of the stimulus. (Whereas repetition suppression mechanisms are generally assumed to result from changes in the dynamics of bottom-up stimulus processing, repetition enhancement has been interpreted as evidence of the involvement of a top-down influence (e.g., [11]–[13], but see [14].) Finally, an *enhanced decision* account, rather than emphasizing perceptual networks, appeals to a specialized “same detection” mechanism whose sensitivity to overlap explains enhanced performance [15].

Each of these accounts of the positional-specificity effect can find physiological plausibility in the existing literature and, importantly for this study, each of these putative physiological correlates is distinct from the others. The *sustained-activity* account is consistent with the fact that early visual areas, including primary visual cortex, represent sample-related information across the delay period (e.g., [16]–[18]). *Repetition suppression* is a well-studied phenomenon observed in many brain areas (e.g., [19], [20]), as is *repetition enhancement* (e.g., [21], [22]). Finally, *enhanced decision* was proposed to account for the results of an event-related potential (ERP) study of the positional-specificity effect, in which “identification of the test stimulus as a target appears to be mainly a left hemispheric process” [15] (p. 425). One goal of the present study, therefore, was to use functional magnetic resonance imaging (fMRI) to test among these four models. Before doing so, however, we performed several behavioral experiments to establish boundary conditions of the positional-specificity effect and to confirm that it can be produced with a procedure that is compatible with event-related fMRI.

## Experiment 1

This experiment established normative values against which the subsequent experiments using these materials and this general procedure could be compared.

### Method

#### Ethics statement

This experiment was approved by the Social and Behavioral Sciences Institutional Review Board (IRB) of the University of California, San Diego (UCSD) Human Research Protections Program.

#### Subjects

Sixteen observers from the University of California, San Diego community participated in a one-hour procedure in exchange for course credit in an introductory psychology class. Each provided written informed consent prior to participating.

#### Materials

The stimuli (presented in white on a black background) were created by randomly filling 7 of 16 cells in a 4 by 4 matrix of squares (3 deg. of visual angle on each side).

#### Procedure

**Figure 1:**
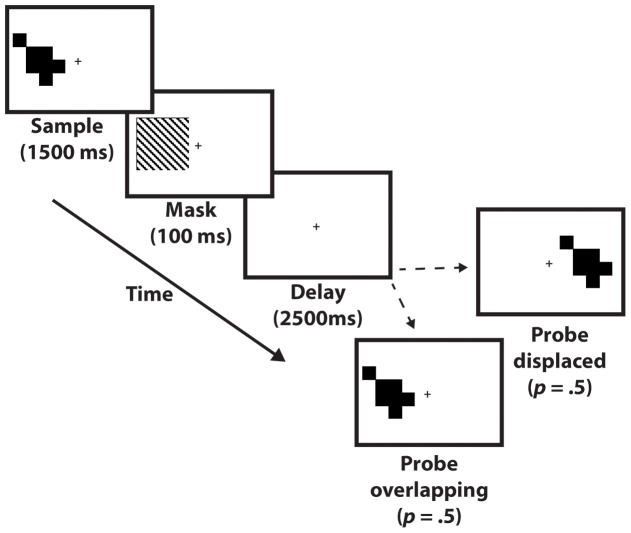
Schematic illustration of the task from Experiment 1.

Observers were seated 18” from the screen where stimuli were presented. All stimuli appeared in one of two locations on the screen, centered on a point on the horizontal meridian 3 deg. of visual angle to the left or right of central fixation. Each trial began with the presentation of a sample stimulus unpredictably to the right or left of fixation for 1500 msec, followed by a mask (100 msec), followed by a delay period (2500 msec), followed by a memory probe stimulus that remained visible until the observer’s response. The probe matched the sample with *p* = .5, and, independently, its location with *p* = .5. Probes that mismatched the sample stimulus were created by randomly selecting a filled cell and moving it to a previously empty location. A central fixation cross was present throughout the trial and observers were instructed to maintain fixation throughout the trial until they responded to the probe stimulus (and, explicitly, not to foveate the sample). (See Figure 1.) Each observer performed a total of 104 trials, broken into four blocks of 26 trials each.

During this and all subsequent behavioral studies (i.e., Experiments 2–4), an experimenter sat next to the screen, faced the observer, and carefully watched the observer’s eyes, to ensure that s/he did not break fixation during the trial. Although procedure does not provide eye-position data, pilot testing confirmed that it was an effective way to detect saccades made to stimulus locations. Additionally, because probe position was equiprobable and unpredictable, the optimal strategy was to maintain central fixation during the delay period, and this was explained to each observer prior to data collection.

### Results and Discussion

**Figure 2:**
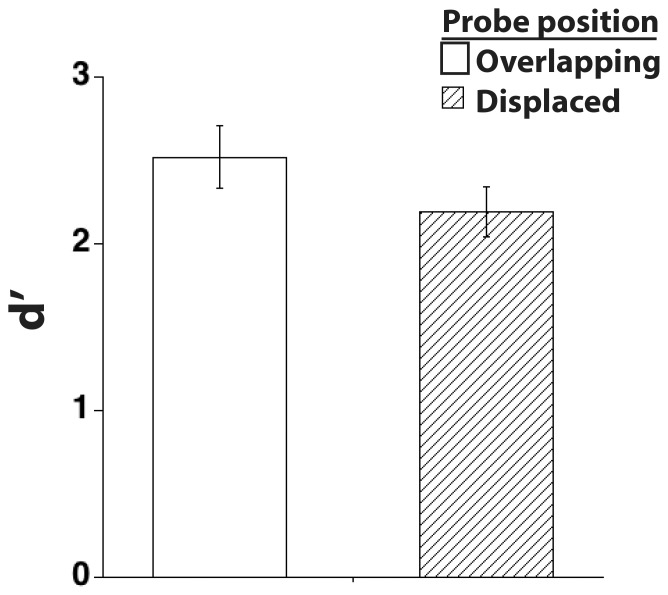
Results from Experiment 1, illustrating improved performance when the position on the screen of the probe overlapped that of the sample – a positional-specificity effect. Errors bars show standard error of the mean, to illustrate the variability in the data.

The results (illustrated in Figure 2) replicated previous demonstrations of the positional-specificity effect. Performance in the memory task was converted into *d’* to examine observers’ sensitivity to the difference between probes that either matched or mismatched the shape of the sample stimulus, as a function of whether the probe was overlapping or displaced relative to the sample position. In line with previous findings, d’ was reliably higher for probes whose position overlapped that of the sample stimulus than for probes that appeared in the opposite visual field (t(15) = 2.2, *p*<.05). No differences in reaction time (RT) were found as a function of whether probe position overlapped (M = 943 ms) or was displaced (M = 938) relative to the position of the sample (*p = *.81), suggesting that a speed accuracy tradeoff was not responsible for the observed differences in d’. This latter finding also added to our confidence that observers complied with instructions to maintain fixation throughout the trial, because if eye position had tended to favor the position of sample presentation, one would expect RTs to be longer for displaced probes.

## Experiment 2

To begin addressing questions of mechanism, Experiment 2 assessed positional-specificity effects in a task that required the storage of two sequentially presented objects in VSTM. This design tested the plausibility of both the *sustained-activity* and the *enhanced-decision* accounts of the positional-specificity effect by examining whether it is sensitive to whether additional objects have to be stored after the presentation of the critical stimulus. With regard to *sustained activity*, if the positional-specificity effect is a direct consequence of active storage in VSTM, then it should be observed for all objects presented in a sub-span array of items. For *enhanced-decision*, one would not expect a “same detection” mechanism to be sensitive to the order of presentation of the repeated item. Therefore, a failure to observe a reliable positional-specificity effect for the first of two items would argue against both the *sustained activity* and *enhanced decision* accounts of the positional-specificity effect.

### Method

#### Ethics statement

This experiment was approved by the Social and Behavioral Sciences IRB of the UCSD Human Research Protections Program.

#### Subjects

Twenty-five observers from the University of California, San Diego community participated in a one hour procedure in exchange for course credit in an introductory psychology class. Each provided written informed consent prior to participating.

#### Materials

Materials were the same as in Experiment 1.

#### Procedure

The procedure was similar to Exp. 1, with the differences that instead of a mask, a second sample item was presented 300 msec after the offset of the first (also for a duration of 1500 msec) and at the same location as the first, and the subsequent delay period was 2 sec in duration. Subjects were instructed to remember both objects as accurately as possible.

#### Design

The design was similar to Exp. 1, with the addition of a third factor, which was whether the probe corresponded to the first or the second sample stimulus.

### Results and Discussion

**Figure 3:**
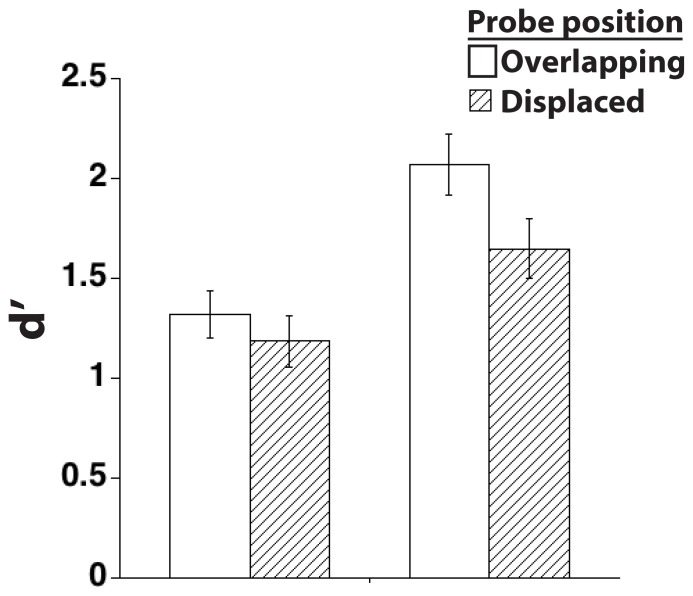
Results from Experiment 2, illustrating that the positional specificity effect is only observed for the most recent of two serially presented sample stimuli. Errors bars show standard error of the mean, to illustrate the variability in the data.

The results indicated that the positional-specificity effect was present for the second of the sequentially presented items in the display (*t*(24) = 3.0, *p*<.01), but not the first (*t*(24) = 1.0; *n.s.*; Figure 3). This difference is best construed as one of degree, however, in that a 2×2 ANOVA revealed main effects of order (first, second: *t*(24) = 26.2, *p*<.0001) and probe position (overlapping, displaced: *t*(24) = 7.7, *p*<.05), but no interaction (*t*(24) = 2.2, *n.s.*). Importantly, memory for the first item presented was well above chance *t*(24) = 12.4, *p*<.0001).

These results indicated that when two objects are presented sequentially, positional-specificity effects are no longer reliable for the first object in the sequence. The results are difficult to reconcile with a *sustained-activity* account, because they suggest that although identity-related information about this first item was presumably actively maintained in VSTM (because this item was recognized at well above chance level), position-related information about it may not have been. They are difficult to reconcile with an *enhanced-decision* account, because a “same detection” mechanism would be expected to be insensitive to the number of items intervening between a sample and its match (e.g., [11]). Thus, by process of elimination, these results appear to be more consistent with passive-trace than with active-maintenance or enhanced-decision accounts of the positional-specificity effect. There were, however, several considerations that impelled us to conduct a follow-up to this experiment. First, because the Order×Position interaction was not reliable, an alternate interpretation of the results remained possible. Specifically, it could be that the position-specificity effect simply scales with the overall strength of the memory trace, and our results merely followed from the fact that d’ for the first item was smaller than for the second item. Second, Experiment 2 confounded item probed (first, second) with the time-in-VSTM. Thus, even if one were to accept at face value the loss of the positional-specificity effect for the first item, one could not know whether this was due to the fact that it had been followed by a second item, or the fact that it had been held in VSTM for a longer period of time than the second item. Third, even if both of these confounds could be ruled out, Experiment 2 did not indicate the nature of processing of the second stimulus that would disrupt the positional-specificity effect for the first stimulus.

## Experiment 3

This experiment addressed the questions and confounds left unresolved by Experiment 2. It did so by presenting three conditions, each with the same duration delay period – “no interference”, “passive interference”, and “active interference”. The first two required no overt response prior to probe onset, and, whereas the second and third presented identical second stimuli, only the third required active processing (i.e., attentional selection, evaluation, and a response (but, importantly, not encoding into VSTM)).

### Method

#### Ethics statement

This experiment was approved by the Social and Behavioral Sciences IRB of the UCSD Human Research Protections Program.

#### Subjects

Eighteen observers from the University of California, San Diego community participated in a one hour procedure in exchange for course credit in an introductory psychology class. Each provided written informed consent prior to participating.

#### Materials

The shape stimuli to be remembered were identical to those used in Experiment 1. In addition, some trials included the presentation of a second object that was either symmetrical or not around its horizontal axis. These objects were created by filling 6 cells in a matrix of cells that was three cells wide and four cells tall. First, three cells were randomly selected to be filled in the top (2×3) half of the matrix. Next, the bottom half of the matrix was filled to create an object that was perfectly symmetrical around the horizontal axis, or one cell was shifted to disrupt this symmetry.

#### Design

During “no interference” blocks, the sample stimulus was presented for 1500 ms, followed by a 3950 ms delay period. Immediately after the delay period, a probe stimulus appeared and remained visible until observers indicated with a keypress whether it matched or did not match the shape of the sample stimulus. During “passive interference” trials of the same trial structure were employed, including the same sample-to-probe SOA, except that a second object (either symmetrical or not around the horizontal axis) was presented 1500 ms after the offset of the sample stimulus. The second object was visible for 100 ms, and no response was required. During “active interference” trials, the same physical displays were presented as in the passive interference blocks, but observers were also required to press a key (during the delay period) to indicate whether or not the second object was symmetrical around the horizontal axis. Trials were blocked by condition with block order counterbalanced across observers.

### Results and Discussion

**Figure 4:**
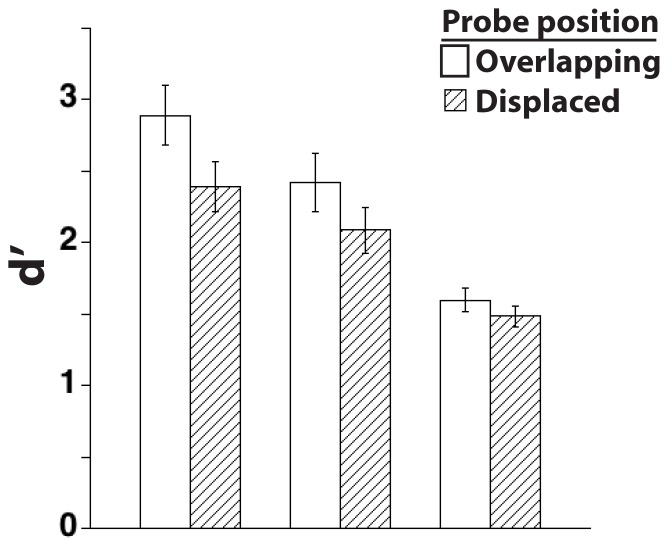
Results from Experiment 3, illustrating disruption of the positional-specificity effect by a subsequently presented item requiring active, but not passive, attentive processing.

As illustrated in Figure 4, the positional-specificity effect for the first item was present in the no-interference (t(17) = 3.6, *p*<.01) and passive-interference (t(17) = 1.7, *p* = .056) conditions, but not in the active-interference condition (t(17) = 1.0, *n.s.*). A repeated measures ANOVA with probe position (overlapping vs. displaced) and interference condition (none, passive, or active) as factors showed a main effects of probe position (F[1], [17] = 12.4, *p*<.01) and of interference condition (F[2], [16] = 25.3, *p*<.0001), but no interaction (F[2], [16] = 2.1, *n.s.*). There was a monotonic decrease in d’, collapsed across probe condition, when comparing performance in the no-interference (d’ = 2.6) vs. passive-interference (d’ = 2.3) vs. active-interference (d’ = 1.5) conditions (F[2], [34] = 28.2, *p*<.01). Planned paired comparisons indicated that although the positional-specificity effect did not differ between no-interference and passive-interference conditions (t(17) = 1.2, *n.s.*), it was reliably larger in the no-interference than in the active-interference condition (t(17) = 2.4, *p*<.05). Thus, these results suggest more strongly than did those of Experiment 2 that the positional-specificity effect is disrupted by attentive processing of an intervening item. Additionally, they confirm that merely lengthening the delay period does not disrupt the effect. Finally, they demonstrate that whereas the mere presentation of an intervening object does not, in and of itself, disrupt the effect, the effect can be disrupted by active processing of the intervening object, even if this processing does not entail volitional encoding into VSTM. This suggests that the positional-specificity effect may not arise from a mnemonic process, per se, but rather may reflect the most recent site of selection.

Taken together, Experiments 1–3 replicate previous demonstrations that probe processing can be enhanced when that stimulus is presented in the same spatial position as the initially stored sample. Novel findings from Experiments 2 and 3, however, indicate that there are conditions under which this positional-specificity effect is not readily observed for items that the behavioral data nonetheless demonstrate to be retained in memory. It follows from this that active retention in VSTM may not be sufficient for episodic spatial information to influence the efficacy of probe processing. This, in turn, calls into question two of the classes of model that we considered in the Introduction: *sustained activity* and *enhanced decision*. What these experiments have not addressed, however, are the two *passive-trace* accounts. This is because neither of these accounts can be assessed directly with a behavioral study. Because both make clear, and distinct, physiological predictions (i.e., an enhanced vs. a decreased probe-evoked response), the remaining two experiments relate to the fMRI study of this task.

## Experiment 4

Prior to conducting an fMRI study of VSTM performance, we needed to verify that the positional-specificity effect would be preserved with the procedure dictated by a “slow” event-related design capable of isolating estimates of fMRI activity evoked by each of the three phases of the task (i.e., sample; delay; probe).

### Method

#### Ethics statement

This experiment was approved by the Biomedical IRB of the University of Pennsylvania.

#### Subjects

Twenty healthy young adults who reported no neurological, psychological, or psychiatric problems were recruited from the University of Pennsylvania community. Prior to participating they provided written informed consent.

#### Materials

Same as Experiment 1.

#### Design and procedure

Same as Experiment 1, with the exceptions that the delay period of each trial was lengthened to 6.5 sec, and that each subject performed a total of 128 trials, broken into four blocks of 32 each.

### Results and Discussion

**Figure 5:**
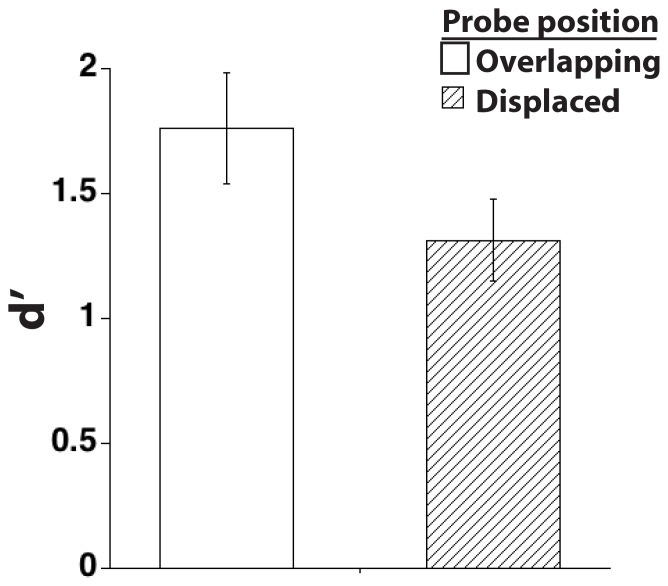
Results from Experiment 4, illustrating a preserved positional-specificity effect when the task is administered with a (fMRI-compatible) delay period of 6.5 sec.

Figure 5 illustrates that overall scores were lower than those from conditions from previous experiments in this report in which a positional-specificity effect was observed, and, instead, were in the range of overall scores from conditions in which the effect was not reliable (i.e., the first item in Experiment 2 (Fig. 3) and the active condition of Experiment 3 (Fig. 4)). Nonetheless, evidence of a preserved positional-specificity effect (*t*(19) = 2.0; *p* = .057) gave us confidence that the effect can be studied with fMRI.

## Experiment 5

The primary rationale behind this study was to effect a test among the two *passive-trace* accounts of the positional-specificity effect: repetition suppression vs. repetition enhancement. This would be accomplished by comparing the magnitude of probe-evoked effects of overlapping probes vs. displaced probes. Additionally, because our method generated discrete estimates of activity from all three trial epochs, we could also confirm the conclusion from Experiments 2 and 3 that neither a bias in sustained delay-period activity nor an enhanced-decision mechanism are likely to account for to the positional-specificity effect. The precise operationalizations of each model’s prediction are listed at the end of the Methods section.

### Method

#### Ethics statement

This experiment was approved by the Biomedical IRB of the University of Pennsylvania and the Health Sciences IRB of the University of Wisconsin-Madison.

#### Subjects

Five healthy young adults participated at the University of Pennsylvania, and four participated at the University of Wisconsin-Madison. All reported no neurological, psychological, or psychiatric problems, and all provided written informed consent in accord with the protocols approved by the relevant university’s Institutional Review Board. The data from all 9 were treated as one sample. Two analyses – the MVPA-based analysis to confirm fixation and the assessment of behavioral data from the scanning sessions – were performed at a later date than the remainder of the analyses described here. (They were performed after peer review of an earlier version of this manuscript.) Due to an archiving error, the data from two subjects were lost during the intervening time, and so these two additional analyses were run on *n* = 7.

#### Behavioral methods

All elements are identical to Exp. 4, with the exception that trials were broken into 8 blocks of 16 trials each, with each block corresponding to an fMRI scan.

#### fMRI data acquisition and analysis

fMRI scanning was performed at two facilities, at the University of Pennsylvania Medical School on a 1.5T scanner (GE SIGNA) and at the Waisman Center for Brain Imaging and Behavior at the University of Wisconsin-Madison on a 3T scanner (GE SIGNA). For all subjects we acquired high-resolution T1-weighted anatomical images, and gradient echo, echoplanar sequences (TR = 2000 ms, TE = 50 ms) were used to acquire data sensitive to the blood oxygen level dependent (BOLD) signal. Scans of the VSTM task were preceded by a scan in which we derived an estimate of the hemodynamic response function (HRF) for each participant [23]. The HRF, which characterizes the fMRI response resulting from a brief impulse of neural activity [24], was used to convolve independent variables entered into the modified general linear model (GLM, [25] that were used to analyze the results of the scans of the VSTM task.

The principle of the fMRI time series analysis was to model the fMRI signal changes occurring during the three discrete periods of the behavioral trials (sample, delay, probe) with covariates comprised of shifted, BOLD impulse response functions [26], [27]. To accomplish this, we first positioned delta functions at times 0, 4, and 8 of the trial, to model the onset of the sample, the middle of the delay period, and the onset of the probe, respectively. Next, for each subject, we convolved these delta functions with that subject’s empirically derived HRF. An alternative approach may have been to model the delay period with a square wave spanning the entire delay period. Our approach, in contrast, was inherently conservative, in that it traded the possibility of missing some early delay-period activity for the certainty that delay-period estimates would not be contaminated by sample-related variance. This was an acceptable trade-off for this experiment, because it would permit detection of delay-period differences predicted by *sustained-activity* accounts, and any such differences would not be subject to the possibility that they resulted from “spill over” of the sample-evoked response, rather than hemispheric bias in the sustained delay-period activity, itself. This was a critical factor for our analyses, because one would, of course, expect a greater sample-evoked response in contralateral vs. ipsilateral regions. Evidence from simulations and from many relevant empirical studies (e.g., [26]–[28]), however, indicates that the least-squares solution of the GLM would assign all sample-evoked variance in the BOLD signal to the sample covariate. Therefore, any laterality differences in delay and/or probe covariates could only be attributed to true differences in the level of activity during these portions of the trial. Between-condition differences in fMRI signal were tested with contrasts of the coefficients associated with the covariates in question. Because the positional-specificity effect, by definition, produces a confound of accuracy by condition, we included all trials in the analysis (i.e., correct and incorrect responses), so as to have an equal number of trials contributing to each cell of the design.

The analysis plan derived from the assumption, shared by both *sustained-activity* and *passive-trace* models, that the positional-specificity effect arises from the activation of the same neural units that were engaged in processing the sample stimulus (see Introduction). It was implemented by generating sample-evoked functional regions of interest (ROIs), then analyzing the level of activity within these functional ROIs during different epochs from the same task. (For a discussion of such a “factorial design” approach, in which one epoch of the experimental task is used to define an ROI (rather than, e.g., a separate functional localizer scan), and then this ROI is interrogated with orthogonal contrasts, see [29]). The analyses proceeded in six steps, the first five performed on an individual subject basis. *First*, we identified the voxels that demonstrated significant sample-evoked activity for each of the two sample types: LVF and RVF (Bonferroni corrected to *p*<.05). *Second*, we created sample-evoked functional ROIs (separately for LVF- and for RVF-sample trials) by grouping these voxels according to brain region of interest (see next paragraph). *Third*, we extracted the spatially averaged time series from each sample-evoked ROI. *Fourth*, we assessed within each functional ROI evidence for laterality biases. The delay period was assayed with the two-tailed contrast [Delay_LVF Sample_–Delay_RVF Sample_]. A resultant *t*-value with a positive sign would indicate a greater LVF-sample than RVF-sample effect; a *t*-value with a negative sign the opposite. For probe-evoked responses, each ROI was evaluated with the contrast [Probe_LVF Sample_–Probe_RVF Sample_] applied to trials for which the probe was presented to that ROI’s visual field. (Thus, for a LVF-sample ROI, for example, this contrast was applied to all trials for which the probe was presented to the LVF, and a *t*-value with a positive sign would indicate a greater response for overlapping than displaced probes, a *t*-value with a negative sign the opposite.) Recall that at this stage there were still two functional ROIs per brain region, one corresponding to LVF sample-evoked voxels, and one corresponding to RVF sample-evoked voxels. The purpose of the *fifth* step, therefore, was to collapse across trials in order to generate a positional-specificity index for each trial epoch (delay and probe) at each brain region. This was accomplished by subtracting RVF ROI values from step #4 from the corresponding LVF ROI values. It was at this fifth step of the analysis that the different models of the positional-specificity effect made differing predictions:

- A positive sum for the delay contrasts would indicate a relatively greater delay-period effect for same- than opposite-visual-field samples, a result that would be consistent with the *sustained-activity* model.
- A negative sum for a probe-related effect would indicate relatively reduced probe-related effects for overlapping than for displaced probes, a result that would be consistent with the repetition-suppression mechanism of the *passive-trace* model.
- A positive sum for a probe-related effect would indicate relatively greater probe-related effects for overlapping than for displaced probes, a result that would be consistent with the repetition-enhancement mechanism of the *passive-trace* model.
- If none of these three scenarios produced reliable effects, we would investigate a non-perceptual basis for the effect. The *enhanced decision* model, for example, predicted greater probe-evoked responses in the left-hemisphere for all matching probes, regardless of the visual field in which either sample or probe was presented.

Finally, in the *sixth* step we performed random effects group analyses by generating group mean positional-specificity indices for each ROI and trial epoch, and noting from the associated 95% confidence intervals whether these differed reliably from 0. (A rationale for employing *t*-values as indices of fMRI effects, and as dependent values in group analyses, is provided elsewhere [27]).

The anatomical ROIs in which we identified voxels with sample-evoked activity were: primary visual cortex (Brodmann's area (BA) 17); extrastriate occipital and temporal areas encompassed by BAs 18, 19, and 37; anterior inferior temporal gyrus (BA 20); the portions of inferior parietal cortex encompassed by BAs 39 and 40; and the portion of superior parietal lobule encompassed by BA 7. We created these ROIs by drawing them onto the “canonical” representation of a brain in Talairach space that is provided in SPM96b, using the atlas of Talairach and Tournoux [30] to confirm our identification of anatomical landmarks, and transforming these ROIs from Talairach space into the native space in which each participant’s data had been acquired by applying an algorithm for 12 parameter affine transformation [31] with non-linear deformations [32].

To verify central fixation during sample presentation, we used a multivariate pattern classification approach, reasoning that a classifier could only successfully discriminate LVF sample presentation from RVF sample presentation if subjects were, in fact, fixating centrally during this epoch. Classification was performed with L2-regularized logistic regression (λ = 25) implemented with the Princeton Multi-Voxel Pattern Analysis (MVPA) toolbox (www.pni.princeton.edu/mvpa/) and custom routines in MATLAB. For each subject a “retinotopic ROI” was created by merging the BA 17, 18, and 19 anatomical ROIs. From this “retinotopic ROI”, for trials on which the subject performed correctly, data from the first volume of each trial (corresponding to Sample presentation) were labeled according to visual field of presentation and used to train the classifier using the leave-one-out cross-validation procedure. That is, for each subject, a classifier was trained on data from all but one trial (mean 81 trials, range 67–95 trials), and then tested on the remaining trial, rotating through all possible permutations. The group-level significance of classifier performance was determined with a two-tailed, paired t-test, testing against chance performance of 0.5.

### Results and Discussion

#### Behavior

The behavioral results demonstrated a positional-specificity effect, with superior performance on overlapping (d’ = 2.29) than on nonoverlapping (d’ = 2.10) trials, although, with this small n, the difference did not achieve statistical significance (*t*(6) = 1.74, n.s.).

#### fMRI

**Figure 6:**
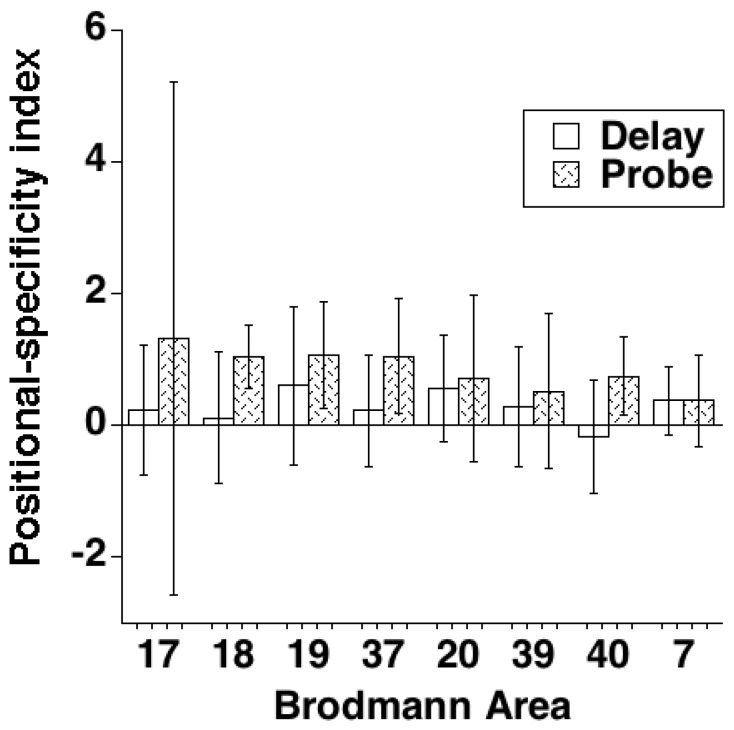
Group results from Experiment 5, by trial epoch and by brain region. Error bars indicate 95% confidence intervals.

Generation of sample-evoked activity maps revealed extensively bilateral (although partly nonoverlapping) patterns of activity for both LVF and RVF samples. Regions of nonoverlap tended to reflect greater contra- than ipsilateral responses to the samples. (The partial exception to this was area 17, for which, in three subjects, no sample-evoked voxels were identified for at least one of the two sample types. This precluded entering area 17 data from these three subjects into the group analyses.) The delay-period and probe-evoked response, by ROI (i.e., the results from step #4 of the 5-step analysis), are illustrated in Table 1. The hypothesis-testing group analyses of positional-specificity indices, illustrated in Figure 6, with accompanying inferential statistics in Table 2, revealed no functional ROI for which delay-period activity was reliably higher for same- than opposite-visual-field samples. They did, however, reveal reliably positive positional-specificity effects for probe-evoked activity in areas 18, 19, 37, and 40. That is, probe-evoked activity in the functional ROIs in these regions was greater on trials when the probe appeared in the same location as had the sample than on trials when the probe appeared in the opposite hemisphere. (For area 17, for which the group trend was not reliable with an *n* of 5, the positional-specificity index was positive for three subjects and negative for two.) Table 3 confirms that effect sizes did not differ systematically across the two scanners. Fixation at the time of sample presentation was confirmed for the seven subjects for whom data were available for this analysis by successful multivariate classification of LVF vs. RVF trials (t (6) = 2.58, *p*<.05).

**Table 1:** Mean delay-period effects [Delay_LVF Sample_–Delay_RVF Sample_] and probe-evoked effects [Probe_LVF Sample_–Probe_RVF Sample_] as a function of visual field of sample presentation (*t* values [SD]).

		ROI (Brodmann Area)															
		17		18		19		37		20		39		40		7	
		RVF sample-evoked	LVF sample-evoked	RVF sample-evoked	LVF sample-evoked	RVF sample-evoked	LVF sample-evoked	RVF sample-evoked	LVF sample-evoked	RVF sample-evoked	LVF sample-evoked	RVF sample-evoked	LVF sample-evoked	RVF sample-evoked	LVF sample-evoked	RVF sample-evoked	LVF sample-evoked
Epoch	Delay	−0.03 [1.10]	0.29 [0.51]	0.13 [1.50]	0.50 [1.37]	0.11 [1.46]	0.89 [1.12]	0.41 [1.23]	0.48 [1.04]	−0.07 [1.34]	0.25 [0.61]	0.52 [1.74]	0.95 [1.05]	0.55 [1.17]	−0.21 [0.76]	0.52 [1.36]	0.75 [1.19]
	Probe	−0.63 [1.68]	1.37 [1.71]	0.95 [1.18]	1.73 [0.92]	0.19 [1.41]	1.38 [1.21]	−0.39 [1.54]	0.56 [1.27]	−0.47 [1.57]	0.14 [0.28]	−0.45 [1.16]	0.49 [0.81]	−0.15 [1.24]	0.52 [0.88]	0.20 [0.91]	0.68 [1.35]

(Note: these data correspond to step #4 of analysis plan; see text.).

**Table 2:** Two-tailed *t* statistics from group analyses of positional-specificity indices [(df) *t*].

	ROI (Brodmann Area)							
	17	18	19	37	20	39	40	7
Delay	(4) 0.548	(6) 0.364	(8) 1.356	(8) 0.738	(6) 1.671	(6) 0.940	(8) 0.557	(7) 1.743
Probe	(5) 1.158	(6) 6.669	(8) 3.5158	(8) 3.214	(7) 1.6188	(6) 1.370	(8) 3.413	(8) 1.492

**Table 3:** Mean positional-specificity effect size (*t* values [*SD*]), by scanner.

epoch	scanner	ROI (Brodmann Area)							
		17	18	19	37	20	39	40	7
Delay	1.5 T	−0.106 [0.229]	−0.407 [1.071]	0.552 [0.936]	0.206 [0.596]	−0.337 [0.500]	0.475 [0.866]	−1.092 [1.193]	0.638 [0.571]
	3 T	0.239 [0.774]	−0.443 [0.823]	−0.202 [0.052]	0.207 [0.454]	0.260 [0.271]	−0.248 [0.346]	0.332 [0.242]	−0.052 [0.251]
Probe	1.5 T	3.039 [2.935]	1.112 [0.572]	1.452 [0.602]	1.210 [1.159]	1.250 [0.982]	1.368 [0.875]	1.018 [0.392]	0.833 [0.418]
	3T	−1.245 [0.286]	0.947 [0.189]	0.442 [1.293]	0.061 [1.534]	0.737 [1.313]	−0.315 [0.463]	0.303 [0.976]	−0.358 [0.857]

These results are broadly consistent with Dill and Fahle’s [1] proposal that the positional-specificity effect is supported, at multiple levels of the visual system, by networks that had participated in the processing of the sample stimulus. Regarding the mechanism underlying the effect, within passive-trace models, the finding of positive effects in probe-evoked activity in extrastriate, temporal, and parietal regions is consistent with a repetition-enhancement account. Further, the absence of reliable positional-specificity of delay-period activity at any functional ROI was consistent with our interpretations of Experiments 2 and 3, and thereby provides an independent source of evidence that fails to support an active-trace account. It should be noted, however, that our estimates of delay-period activity, although not statistically higher for same- than opposite-visual-field samples in any ROI, were nonetheless numerically biased in this direction for all ROIs except area 40. Thus, we cannot rule out the possibility that the positional-specificity effect may receive some relatively weak support from biased delay-period activity that is congruent with that quantitatively (and significantly) larger probe-evoked effect.

At first glance, it may seem surprising that our estimates of delay-period activity were not biased by visual field in which the sample had been presented. This implies, for example, that for trials in which the sample was presented in the right visual field, in no brain area were estimates of delay period activity higher in RVF than in LVF ROIs. Several factors, however, help to explain this result. First, although it was almost certainly the case that, for example, samples presented in the right visual field produced a greater evoked response in RVF than LVF ROIs (see two illustrations of this in Fig. 5), any resultant “spillover” into early portions of the delay period would not be expected to affect our estimates of delay-period activity, because our procedure was expressly designed to yield estimates of delay-period activity that are not contaminated by sample-evoked signal [26], [27]. Second, it is unlikely that the finding of null effects for the delay period but positive effects for memory probes merely reflects reduced sensitivity of measurements of the former, because, with virtually the same experimental procedures, we have previously found reliable hemispheric biases in delay-period activity when subjects were performing delayed recognition of the location of sample stimuli [33]. In the present study, in contrast, sample location was not related to the validity of the probe. Finally, in a VSTM task that resembled the present task in that sample stimuli were presented to just one visual field, Ester et al. [34] have demonstrated with MVPA that voxels located ipsilateral to the visual field of sample presentation can carry a delay-period representation of the sample that does not differ in strength (of classification accuracy) from the representation supported by contralateral voxels. Thus, the delay-period of VSTM tasks that do not explicitly require memory for location may be characterized by spatially global (i.e., not just retinotopic) recruitment of neurons that represent critical features of the sample stimulus.

## General Discussion

Taken as a whole, our results point to a passive-trace account of the positional-specificity effect. Experiments 2 and 3 indicated that the positional-specificity effect is greatly attenuated, if not abolished, when an attention-demanding item is presented in between the to-be-remembered sample and the recognition probe. These results are difficult to reconcile with a sustained-activity account, because in neither experiment did the insertion of a distractor abolish overall memory for the sample. The fMRI results were consistent with this interpretation of the behavioral results, in that they did not reveal any reliable positional-specificity of delay-period activity. They did, however, provide strong positive evidence for a passive-trace account by showing that the positional-specificity effect is associated with an increase in the probe-evoked response (i.e., repetition enhancement). These results therefore suggest that the act of selecting an item for attentional processing leaves a tag that enhances the processing of a subsequent item if that item is presented at the same retinotopic location.

One possible concern about our interpretation of the behavioral results is that manipulations that disrupted the positional-specificity effect also tended to reduce overall memory performance. If it were the case that the size of this effect simply scales with task difficulty, this would leave open the possibility that task-irrelevant spatial information is carried as part of the active representation in VSTM, but it simply drops below the statistical threshold when overall accuracy is low. We believe, however, that integrating across experiments rules out this alternative: In Experiment 3, the positional-specificity effect in the active-interference condition was not only no longer reliable vs. baseline, it was also significantly smaller than in the no-interference condition; in Experiment 4 the effect was preserved despite an overall level of performance that, in the previous two experiments, was associated with the loss of the effect; and in Experiment 5, there was no strong neural evidence for a laterality bias in delay-period activity, despite significantly elevated delay-period activity.

Repetition effects are commonly observed in physiological studies of perception and memory. In long-term memory (LTM) research, repetition suppression is the effect most commonly associated with repetition priming with familiar stimuli (e.g., [19], [22], [35]). Repetition enhancement is seen less often, and may depend on such factors as the familiarity of the stimuli [22] and the visual quality with which they are presented [36]. Both repetition suppression and repetition enhancement have been observed with explicit object recognition (e.g., [37], [38]). The cellular basis of repetition effects observed with fMRI remains a matter of debate [20]. In short-term recognition, both repetition suppression and repetition enhancement have been observed with the ABBA task, in which a monkey views a series of stimuli and is rewarded for responding to a repetition of the trial-initial target (“A”) but not for responding to repetitions of foils (“B”) – the former producing repetition enhancement and the latter repetition suppression [11], [12]. Because neurons displaying repetition enhancement are more prevalent in prefrontal than inferotemporal (IT) cortex [12], and because repetition suppression in IT has also been observed absent any explicit task (e.g., to repeated stimuli presented during passive fixation and even while the animal was under anaesthesia, [39]–[41]), Miller and colleagues (1994, 1996) have proposed that the former reflects an active working memory process (“match enhancement”), and the latter the automatic signaling of repetition, regardless of behavioral relevance. Repetition enhancement vs. suppression have also been described in a human fMRI study in which VSTM is combined with visual search [13]. Within one set of regions (superior frontal and inferior temporal), if the initial presentation of a shape was as the sample in a VSTM task (with the search being a second task occurring during the delay period), the outcome was repetition enhancement; if the initial presentation was as an item that required a perceptual judgment but that did not need to be held in VSTM (and thus produced “mere repetition”), the outcome was repetition suppression. In these regions, repetition enhancement was observed regardless of whether the repeating shape validly or invalidly cued the search target, even though this distinction had a significant effect on search times. In a second set of regions (prefrontal and thalamic), only on VSTM trials, enhancement occurred when the repeating shape validly cued the search target and suppression on invalid cuing trials. In the first set of regions, the authors attributed the opposite repetition effects associated with VSTM vs. mere repetition as reflecting a top-down influence on visual selection vs. a bottom-up change in visual processing efficiency (i.e., repetition priming). To the set of regions sensitive to cuing validity, the authors ascribed a monitoring function that produced opposing effects depending on the congruence of the stimulus relative to the goals of the concurrent VSTM and visual search tasks.

Despite the existence of this extensive literature on repetition effects, the present results do not map cleanly onto any one of the phenomena summarized here. Although it has a top-down element in that it is observed only at the most recent site of attentional selection, other effects sharing this property either depend on probe validity (i.e., match-enhancement [12] and the valid-cue effect [13]) or do not demonstrate positional specificity (the VSTM-vs.-mere-repetition effect [13]). (Note that our repetition-enhancement effect was observed independent of validity – for example, half of the nonoverlapping probes in our study were valid probes). With regard to repetition priming, although the positional specificity-effect shares the quality of reflecting the influence of a prior task-irrelevant event on stimulus processing, most repetition priming effects with which we are familiar have not been shown to be retinotopically specific. Additionally, repetition priming effects can be observed over long lags during which many other items are presented [42], whereas Experiments 2 and 3 showed that the positional-specificity effect is highly sensitive to interference. Nonetheless, there are examples of repetition priming associated with repetition enhancement. These tend to be when stimuli are novel (e.g., [22]), and Henson has proposed that repetition enhancement may occur when the subsequent presentation of an item recruits new processes that were not engaged by the initial presentation, as might happen when a representation of a previously novel stimulus is being formed [37]. Although behavioral studies indicate that the positional-specificity effect is only seen with novel, nonrepresentational stimuli, it will be important in future neuroimaging studies to compare directly how the factors of novelty and sample-to-probe overlap interact.

At an abstracted level, the positional-specificity effect indicates that VSTM shares the property with LTM that task-irrelevant context at the time of encoding can influence subsequent recognition, and that the retention of this information does not appear to depend on an active process. Of course, the sensitivity of the positional-specificity effect to retroactive interference, among other features, indicates that the nature of the contextual codes that influence VSTM and LTM are quite different. Nonetheless, our results represent another example of the fact that the evaluation of the recognition probe can recruit similar processes in these two types of memory task (e.g., [43]–[46]). They also indicate that at least some of the information that is retained in VSTM tasks is stored in a passive trace that is reactivated at the time of the memory decision.

## References

[1] Dill M, Fahle M (1998) Limited translation invariance of human visual pattern recognition. Perception & Psychophysics 60: 65–81. 9503912 10.3758/bf03211918

[2] Foster DH, Kahn JI (1985) Internal representations and operations in the visual comparison of transformed patterns: effects of pattern point-inversion, position symmetry, and separation. Biological Cybernetics 51: 305–312. 3978145 10.1007/BF00336917

[3] Dill M, Fahle M (1999) Display symmetry affects positional specificity in same-different judgment of pairs of novel visual patterns. Vision Research 39: 3752–3760. 10746146 10.1016/s0042-6989(99)00068-1

[4] Fuster JM (1995) Memory in the Cerebral Cortex. Cambridge, MA: MIT Press.

[5] Goldman-Rakic PS (1987) Circuitry of the prefrontal cortex and the regulation of behavior by representational memory. In: Mountcastle VB, Plum F, Geiger SR, editors. Handbook of Neurobiology. Bethesda: American Physiological Society. 373–417.

[6] Curtis CE, D'Esposito M (2003) Persistent activity in the prefrontal cortex during working memory. Trends in Cognitive Sciences 7: 415–423. 12963473 10.1016/s1364-6613(03)00197-9

[7] Vogel EK, Machizawa MG (2004) Neural activity predicts individual differences in visual working memory capacity. Nature 428: 748–751. 15085132 10.1038/nature02447

[8] Johnson JS, Spencer JP, Luck SJ, Schöner G (2013) A dynamic neural field model of visual working memory and change detection. Psychological Science. In press. 10.1111/j.1467-9280.2009.02329.x PMC2782451 19368698

[9] Mazaheri A, Jensen O (2008) Asymmetric amplitude modulations of brain oscillations generate slow evoked responses. The Journal of Neuroscience 28: 7781–7787. 18667610 10.1523/JNEUROSCI.1631-08.2008 PMC6670375

[10] van Dijk H, van der Werf J, Mazaheri A, Medendorp WP, Jensen O (2010) Modulations of oscillatory activity with amplitude asymmetry can produce cognitively relevant event-related responses. Proceedings of the National Academy of Science (USA) 107: 900–905. 10.1073/pnas.0908821107 PMC2818898 20080773

[11] Miller EK, Desimone R (1994) Parallel neuronal mechanisms for short-term memory. Science 263: 520–522. 8290960 10.1126/science.8290960

[12] Miller EK, Erickson CA, Desimone R (1996) Neural mechanisms of visual working memory in prefrontal cortex of the Macaque. Journal of Neuroscience 16: 5154–5167. 8756444 10.1523/JNEUROSCI.16-16-05154.1996 PMC6579322

[13] Soto D, Humphreys GW, Rotshtein P (2007) Dissociating the neural mechanisms of memory-based guidance of visual selection. Proceedings of the National Academy of Science (USA) 104: 17186–17191. 10.1073/pnas.0703706104 PMC2040391 17940037

[14] Ewbank MP, Lawson RP, Henson RN, Rowe JB, Passamonti L, et al (2011) Changes in “top-down” connectivity underlie repetition suppression in the ventral visual pathway. The Journal of Neuroscience 31: 5635–5642. 21490204 10.1523/JNEUROSCI.5013-10.2011 PMC3759805

[15] Talsma D, Wijers AA, Klaver P, Mulder G (2001) Working memory processes show different degrees of lateralization: Evidence from event-related potentials. Psychphysiology 38: 425–439. 11352131

[16] Serences JT, Ester EF, Vogel EK, Awh E (2009) Stimulus-specific delay activity in human primary visual cortex. Psychological Science 20: 207–214. 19170936 10.1111/j.1467-9280.2009.02276.x PMC2875116

[17] Harrison SA, Tong F (2009) Decoding reveals the contents of visual working memory in early visual areas. Nature 458: 632–635. 19225460 10.1038/nature07832 PMC2709809

[18] Super H, Spekreijse H, Lamme VAF (2001) A neural correlate of working memory in the monkey primary visual cortex. Science 293: 120–124. 11441187 10.1126/science.1060496

[19] Schacter DL, Buckner RL (1998) Priming and the brain. Neuron 20: 185–195. 9491981 10.1016/s0896-6273(00)80448-1

[20] Grill-Spector K, Henson R, Martin A (2006) Repetition and the brain: neural models of stimulus-specific effects. Trends in Cognitive Sciences 10: 14–23. 16321563 10.1016/j.tics.2005.11.006

[21] Miller EK, Li L, Desimone R (1991) A neural mechanism for working and recognition memory in inferior temporal cortex. Science 254: 1377–1379. 1962197 10.1126/science.1962197

[22] Henson RNA, Shallice T, Dolan R (2000) Neuroimaging evidence for dissociable forms of repetition priming. Science 287: 1269–1272. 10678834 10.1126/science.287.5456.1269

[23] Aguirre GK, Zarahn E, D’Esposito M (1998) The variability of human, BOLD hemodynamic responses. NeuroImage 8: 360–369. 9811554 10.1006/nimg.1998.0369

[24] Boynton GM, Engel SA, Glover GH, Heeger DJ (1996) Linear systems analysis of functional magnetic resonance imaging in human V1. The Journal of Neuroscience 16: 4207–4221. 8753882 10.1523/JNEUROSCI.16-13-04207.1996 PMC6579007

[25] Worsley KJ, Friston KJ (1995) Analysis of fMRI time-series revisited–again. NeuroImage 2: 173–182. 9343600 10.1006/nimg.1995.1023

[26] Zarahn E, Aguirre GK, D'Esposito M (1997) A trial-based experimental design for fMRI. NeuroImage 6: 122–138. 9299386 10.1006/nimg.1997.0279

[27] Postle BR, Zarahn E, D'Esposito M (2000) Using event-related fMRI to assess delay-period activity during performance of spatial and nonspatial working memory tasks. Brain Research Protocols 5: 57–66. 10719266 10.1016/s1385-299x(99)00053-7

[28] Postle BR (2005) Analysis of fMRI data from tasks containing temporal dependencies: An evaluation of Slotnick (2005). Cognitive Neuropsychology 22: 921–924. 21038283 10.1080/02643290442000464

[29] Friston KJ, Rotshtein P, Geng JJ, Sterzer P, Henson RN (2006) A critique of functional localisers. NeuroImage 30: 1077–1087. 16635579 10.1016/j.neuroimage.2005.08.012

[30] Talairach J, Tournoux P (1988) Co-Planer Stereotaxic Atlas of the Human Brain. New York: Thieme Medical Publishers.

[31] Friston KJ, Ashburner J, Frith CD, Poline J-B, Heather JD, et al (1995) Spatial registration and normalization of images. Human Brain Mapping 2: 165–189.

[32] Ashburner J, Friston K (1996) Fully three-dimensional nonlinear spatial normalization: a new approach. NeuroImage 3: S111.

[33] Postle BR, Awh E, Jonides J, Smith EE, D'Esposito M (2004) The where and how of attention-based rehearsal in spatial working memory. Cognitive Brain Research 20: 194–205. 15183391 10.1016/j.cogbrainres.2004.02.008

[34] Ester EF, Serences JT, Awh E (2009) Spatially global representations in human primary visual cortex during working memory maintenance. The Journal of Neuroscience 29: 15258–15265. 19955378 10.1523/JNEUROSCI.4388-09.2009 PMC2830793

[35] Buckner RL, Goodman J, Burock M, Rotte M, Koustaal W, et al (1998) Functional-anatomic correlates of object priming in humans revealed by rapid presentation event-related fMRI. Neuron 20: 285–296. 9491989 10.1016/s0896-6273(00)80456-0

[36] Turk-Browne NB, Yi D–J, Leber AB, Chun MM (2007) Visual quality determines the direction of neural repetition effects. Cerebral Cortex 17: 425–433. 16565294 10.1093/cercor/bhj159

[37] Henson RNA (2003) Neuroimaging studies of priming. Progress in Neurobiology 70: 53–81. 12927334 10.1016/s0301-0082(03)00086-8

[38] Brown MW, Aggleton JP (2001) Recognition memory: what are the roles of the perirhinal cortex and hippocampus? Nature Reviews Neuroscience 2: 51–61. 11253359 10.1038/35049064

[39] Miller EK, Gochin PM, Gross CG (1991) Habituation-like decrease in the responses of neurons in inferior temporal cortex of the macaque. Visual Neuroscience 7: 357–362. 1751421 10.1017/s0952523800004843

[40] Riches IP, Wilson FAW, Brown MW (1991) The effects of visual stimulation and memory on neurons of the hippocampal formation and the neighboring parahippocampal gyrus and inferior temporal cortex of the primate. The Journal of Neuroscience 11: 1763–1779. 2045886 10.1523/JNEUROSCI.11-06-01763.1991 PMC6575394

[41] Vogels R, Sary G, Orban GA (1995) How task-related are the responses of inferior temporal neurons? Visual Neuroscience 12: 207–214. 7786842 10.1017/s0952523800007884

[42] Henson RN, Goshen-Gottstein Y, Ganel T, Otten LJ, Quayle A, et al (2003) Electrophysiological and haemodynamic correlates of face perception, recognition and priming. Cerebral Cortex 13: 793–805. 12816895 10.1093/cercor/13.7.793

[43] Sternberg S (1966) High-speed scanning in human memory. Science 153: 652–654. 5939936 10.1126/science.153.3736.652

[44] Ratcliff R (1985) Theoretical interpretations of the speed and accuracy of positive and negative responses. Psychological Review 92: 212–225. 3991839

[45] Proctor RW (1986) Response bias, criteria settings, and the fast-same phenomenon: A reply to Ratcliff. Psychological Review 93: 473–477.

[46] Awh E, Barton B, Vogel EK (2007) Visual working memory represents a fixed number of items, regardless of complexity. Psychological Science 18: 622–628. 17614871 10.1111/j.1467-9280.2007.01949.x

